# Dysmegakaryopoiesis and Transient Mild Increase in Bone Marrow Blasts in Patients With Aplastic Anemia Treated With Eltrombopag May Be Signs of Hematologic Improvement and Not Portend Clonal Evolution

**DOI:** 10.1093/ajcp/aqac094

**Published:** 2022-08-26

**Authors:** Akira Matsuda, Kazunori Imada, Naoshi Obara, Hiroatsu Iida, Hirohito Yamazaki, Yoshiaki Tomiyama, Koichi Miyamura, Osamu Sasaki, Tetsuo Maeda, Kensuke Ohta, Kensuke Usuki, Yukihiro Tokumine, Kenji Imajo, Yuji Okamoto, Mami Murakami, Shinji Nakao

**Affiliations:** Department of Hemato-Oncology and Medical Education, Saitama International Medical Center, Saitama Medical University, SaitamaJapan; Department of Hematology, Japanese Red Cross Osaka Hospital, Osaka, Japan; Department of Hematology, University of Tsukuba, Tsukuba, Japan; Department of Hematology, National Hospital Organization Nagoya Medical Center, Nagoya, Japan; Division of Transfusion Medicine, Kanazawa University Hospital, Kanazawa, Japan; Department of Hematology and Oncology, Osaka University Hospital, Osaka, Japan; Department of Hematology, Japanese Red Cross Nagoya Daiichi Hospital, Nagoya, Japan; Department of Hematology, Miyagi Cancer Center, Natori, Japan; Department of Hematology, Suita Municipal Hospital, Suita, Japan; Hematology Ohta Clinic, Shinsaibashi, Japan; Department of Hematology, NTT Medical Center Tokyo, Tokyo, Japan; Department of Hematology, Itami City Hospital, Itami, Japan; Department of Hematology, Okayama City Hospital, Okayama, Japan; Novartis Pharma, Tokyo, Japan; Novartis Pharma, Tokyo, Japan; Kanazawa University Institute of Medical Pharmaceutical and Health Sciences, Kanazawa, Japan

**Keywords:** Aplastic anemia, Blast count, Bone marrow, Eltrombopag, Hematologic improvement, Dysmegakaryopoiesis

## Abstract

**Objectives:**

Eltrombopag, a thrombopoietin-receptor agonist, stimulates hematopoiesis in patients with acquired aplastic anemia (AA). Cytomorphologic changes in bone marrow after eltrombopag administration are still unclear. This study examined the effect of eltrombopag on cytomorphologic findings using data from prior phase 2 studies (E1201 and E1202).

**Methods:**

Microscopic examinations were performed in 31 patients with AA (E1201 [n = 21], E1202 [n = 10]). The relationship between hematologic improvement and morphologic findings was also investigated.

**Results:**

In 5 patients (E1201 [n = 3], E1202 [n = 2]), the bone marrow blast count increased after initiation of eltrombopag treatment compared with screening values. The blast count was less than 5%, and the increase in bone marrow blasts was transient in all 4 patients who had bone marrow examinations at follow-up. In 8 patients (E1201 [n = 5], E1202 [n = 3]), dysplastic forms of megakaryocytes were found in the bone marrow following treatment initiation. Dysmegakaryopoiesis of 10% or more was found in 3 patients. None of the patients revealed micromegakaryocytes. Ten patients showed an increase in bone marrow blasts and/or dysmegakaryopoiesis following treatment initiation. Nine of 10 patients showed hematologic improvement in 1 or more lineages.

**Conclusions:**

Dysmegakaryopoiesis without micromegakaryocytes and a transient increase of less than 5% in bone marrow blast count may be signs of hematologic improvement with eltrombopag for patients with AA.

Key Points

- Clonal evolution after eltrombopag treatment in patients with aplastic anemia (AA) is reported, but cytomorphologic changes in bone marrow during administration are unknown.
- Dysmegakaryopoiesis without micromegakaryocytes and a transient increase of <5% in bone marrow blast count may be a marker of hematologic improvement following eltrombopag treatment in patients with AA.
- Even if these findings are confirmed in AA patients receiving eltrombopag therapy, they may not be progressing to myeloid neoplasms.

## INTRODUCTION

Acquired aplastic anemia (AA) is a rare bone marrow failure disorder often characterized by pancytopenia and hypoplastic bone marrow. It occurs through immune-mediated suppression of hematopoiesis and results in the reduction of hematopoietic stem cells (HSCs).^1-3^ Immunosuppressive therapy (IST) with antithymocyte globulin (ATG) and cyclosporine A (CsA) has been the standard treatment option for patients with AA who are not eligible for allogeneic HSC transplantation or lack a matched sibling donor.^4^ Nearly 60% to 70% of patients show a hematologic response rate, but almost one-third of these patients with AA experience a relapse or develop treatment resistance within 2 years.^5,6^

The interaction of eltrombopag, a thrombopoietin (TPO) mimetic, with a TPO receptor on the myeloproliferative leukemia protein leads to the differentiation of HSCs to megakaryocytes.^7,8^ Preclinical data have shown that eltrombopag promoted multilineage hematopoiesis through the expansion of bone marrow HSCs or hematopoietic progenitor cells of human umbilical cord blood CD34-positive cells in nonobese diabetic/severe combined immunodeficient mice.^8^ Clinical studies have demonstrated the efficacy of eltrombopag in non–East Asian patients with severe AA (SAA) refractory to IST, with a 40% hematologic response in at least 1 lineage at 3 to 4 months.^9, 10^ The combination of eltrombopag with standard IST as the first-line treatment in patients with SAA showed about a 90% overall response rate (ORR) and about a 40% complete response (CR) rate at 6 months, which were higher than historically observed with IST alone (ORR, 66%; CR, 10%).^11^ The phase 2 studies E1201^12^ and E1202^13^ reported pharmacokinetic, efficacy, and safety data for eltrombopag in combination with rabbit ATG/CsA in Japanese patients with AA. In the E1201 study, nearly 50% of patients with AA who were refractory or intolerant to IST achieved hematologic responses in at least 1 lineage at 6 months after the start of treatment, while 5 patients achieved bilineage responses and 1 patient achieved trilineage responses.^12^ The E1202 study showed a 57% and 100% ORR at week 26 in IST-naive patients with SAA and nonsevere AA (NSAA), respectively.^13^ These studies showed that eltrombopag was well tolerated in Japanese patients, with a manageable safety profile up to a maximum dose of 100 mg. That said, the studies suggest more cautious dose adjustments in Japanese patients compared with White patients in view of the potential risk for cytogenetic abnormalities and liver toxicity in patients with East Asian ethnicity.^12,13^

Approximately 15% to 20% of patients with AA develop myelodysplastic syndromes (MDS) or acute myelogenous leukemia (AML) during the natural course of the disease.^14,15^ In practice, AA and low-risk MDS are often misdiagnosed because hematologists rely primarily on morphologic findings and diagnostic criteria that are not mutually exclusive to differentiate these syndromes. According to the 2016 World Health Organization (WHO) classification of MDS, bone marrow features single or multilineage dysplasia. Myeloblasts in bone marrow increase, and by definition the excess blast count ranges between 5% and 19%.^16^ Few studies have reported that clonal evolution or cytogenetic abnormality is associated with dysplasia consistent with MDS and AML, including the loss of chromosome 7 after eltrombopag treatment in patients with AA.^17,18^ Therefore, the influence of eltrombopag on the morphology of bone marrow cells in patients with AA is important. Until now, no detailed reports have been published on the cytomorphologic changes in bone marrow during eltrombopag administration. This study examined the cytomorphologic changes in bone marrow in patients treated with eltrombopag during the E1201 and E1202 studies.

## MATERIALS AND METHODS

### Study Design

This study evaluated the cytomorphologic changes in bone marrow following treatment with eltrombopag (Revolade) in Japanese patients with moderate (stage II) or more severe AA.^12,13^ Patients aged 18 to 80 years with NSAA/SAA (according to the diagnosis criteria established by the Study Group on Idiopathic Hematopoietic Disorder)^19^ who had an Eastern Cooperative Oncology Group performance status of 0 or 1 and adequate organ function were enrolled in 2 nonrandomized, open-label phase 2 studies (E1201 and E1202).^12,13^ Patients who were refractory to ATG-based IST were enrolled in the E1201 study, and those who had not received prior ATG-based IST were enrolled in the E1202 study. Study protocols and amendments were reviewed and approved by the institutional review boards at each study center. All patients provided written consent before study entry. The studies were conducted in accordance with the Declaration of Helsinki.

### Treatment

**Figure 1:**
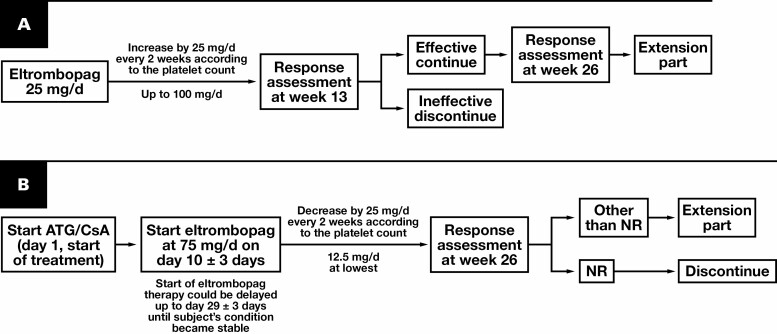
Study design. **A**, E1201 study. **B**, E1202 study. Day 1 was defined as the day when treatment with antithymocyte globulin (ATG)/cyclosporine A (CsA) was started. NR, no response.

In the E1201 study, eltrombopag was initiated at 25 mg once daily and increased by 25 mg per day every 2 weeks up to 100 mg per day to maintain a platelet count between 50 × 10^9^/L and 100 × 10^9^/L. In the extension phase, the eltrombopag dosage was reduced by 50% in patients who achieved a durable response Figure 1A.

In the E1202 study, patients received intravenous rabbit ATG (2.5-3.75 mg/kg/d per local practice) from day 1 to day 5 and oral CsA (3 mg/kg) twice daily on day 1 until week 26. Eltrombopag 75 mg was initiated on day 15. Patients in whom the treatment was assessed as effective (achieved CR/partial response or showed a trend of hematologic improvement) at week 26 entered the extension phase to continue treatment until the product became commercially available Figure 1B.

In both studies, all doses were administered orally in the fasting state. Details of dosage adjustment and monitoring are described in prior publications.^12,13^

### Response Criteria

The response criteria for the E1201 and E1202 studies were assessed based on the established response criteria, as described in prior publications^12,13^Table 1.

**Table 1:** Response Criteria for the E1201 and E1202 Studies

E1201 Study	
Hematologic response rate	Proportion of patients who met at least 1 of the following hematologic response criteria at 6 mo from the start of eltrombopag treatment
Pl-R	Patients in whom the platelet count increased to 20 × 10^9^/L above baseline or transfused patients who achieved transfusion independence for ≥8 wk
Er-R	Nontransfused patients who achieved an increase of 15 g/L in hemoglobin levels from a baseline level of <90 g/L or transfused patients who achieved an absolute reduction of ≥4 units of RBC transfusions for 8 consecutive wk compared with the number of transfusions in the 8 wk before treatment
Ne-R	Patients with a 2-fold increase in neutrophil counts from a baseline level of <0.5 × 10^9^/L or an increase in neutrophil counts of ≥0.5 × 10^9^/L from baseline

E1202 Study		
**Response**	**Patients With Severe/Very Severe AA**	**Patients With Moderate/Moderately Severe AA**
No response	Aggravated or remains severe/very severe	Aggravated/not meeting the criteria mentioned for PR and CR
PR	Transfusion independence (RBC and platelet) and not meeting the criteria for SAA	Transfusion independence (RBC and platelet, when dependent at baseline) or doubling from baseline or normalization of at least 1 blood cell lineage or increase in baseline hemoglobin >30 g/L (when <60 g/L at baseline) or increase in baseline neutrophil count >0.5 × 10^9^/L (when <0.5 × 10^9^/L at baseline) or increase in baseline platelet count >20 × 10^9^/L (when <20 × 10^9^/L at baseline)
CR	Transfusion independence and hemoglobin ≥120 g/L for women and ≥130 g/L for men and absolute neutrophil count ≥1.5 × 10^9^/L and platelet count ≥150 × 10^9^/L	

AA, aplastic anemia; CR, complete response; Er-R, erythroid response; Ne-R, neutrophil response; Pl-R, platelet response; PR, partial response; SAA, severe aplastic anemia.

### Morphologic Analyses of Peripheral Blood/Bone Marrow Examination

In the E1201 study, morphologic assessments using bone marrow aspirations were conducted at screening and twice after initiation of eltrombopag treatment (weeks 13 and 26) or after 7 days of early withdrawal of eltrombopag treatment. During the extension phase, the analyses were performed once every 6 months (ie, weeks 52 and 78), after 7 days of cessation of eltrombopag treatment or end of treatment, and at week 26 during follow-up. During the treatment phase, peripheral blood analyses were conducted at screening and at day 1 (before dosing), every week until dose stabilization (weeks 1, 2, 3, and 4), every 4 weeks after dose stabilization (weeks 9, 13, 17, 21, and 26), after initiation of eltrombopag treatment, after 7 days of cessation of eltrombopag treatment, and during follow-up (at weeks 1, 2, 3, and 4). During the extension phase, peripheral blood analyses were repeated every 4 weeks (ie, weeks 31 and 35) in the administration period, after 7 days of cessation of eltrombopag treatment or end of treatment, and during follow-up (at weeks 1, 2, 3, and 4).

In the E1202 study, in patients who began eltrombopag, the morphologic assessments using bone marrow aspirations were conducted at screening and twice after the start of eltrombopag treatment (weeks 14 and 26) or after 7 days of early withdrawal of eltrombopag. During the extension phase, the analyses were performed once every 6 months (ie, weeks 52 and 78), after 7 days of the cessation of eltrombopag treatment or end of treatment, and during follow-up (at week 26).

Peripheral blood analyses at local centers were conducted at screening and at day 1, every week until week 26 after initiation of treatment, after 7 days of cessation of eltrombopag treatment, and during follow-up (at weeks 1, 2, 3, 4, and 26). Peripheral blood analyses and morphology were reviewed at the central level.

Microscopic examinations of smear were performed using standard methods (peripheral blood: May-Grünwald-Giemsa [MGG] stain; bone marrow: MGG stain and Prussian blue stain). An increase in bone marrow blasts was defined as a bone marrow blast count more than double that at the time of screening. If the blasts were less than 1.0% at the time of screening, the increase in bone marrow blasts was defined as more than 2%. Blast count in the bone marrow was determined using a 500-cell differential of all nucleated cells in a smear. In the peripheral blood, a 200-leukocyte differential was used. The dysplasias were evaluated according to the WHO classification criteria. A minimum of 30 megakaryocytes, 200 erythroblasts, and 200 neutrophils in bone marrow were examined by using 2 smears from each patient. The cutoff levels of each lineage were defined as 10% according to the WHO classification.^16^ Evaluations of the bone marrow cellularity and the number of megakaryocytes were performed semiquantitatively using the specimens of bone marrow trephine biopsy or clot section. Evaluation of the bone marrow cellularity was performed by using low-power fields based on reference standards for bone marrow cellularity.^20^ The normal bone marrow cellularity was defined as 30% to 60%. The bone marrow cellularity was divided into 7 categories: marked hypoplasia (<5%), hypoplasia (5%-19%), slight hypoplasia (20%-29%), normoplasia (30%-59%), slight hyperplasia (60%-69%), hyperplasia (70%-79%), and marked hyperplasia (≥80%). Evaluation of the number of megakaryocytes was performed by using high-power fields (HPFs) (ie, ×400). With reference to the mean number of megakaryocytes reported by Singal and Belliveau,^21^ the normal number of megakaryocytes was defined as approximately 2 per HPF. The number of megakaryocytes was divided into 7 categories: marked decrease (no megakaryocytes), decrease (<0.5/HPF), slight decrease (0.5-0.9/HPF), normal (1.0-2.4/HPF), slight increase (2.5-2.9/HPF), increase (3.0-5.9/HPF), and marked increase (≥6/HPF).

### Chromosome and Fluorescence in Situ Hybridization Analysis

Chromosome analysis and fluorescence in situ hybridization (FISH) analysis for monosomy of chromosome 7 were performed at the time of all bone marrow examinations.

## RESULTS

Overall, data from 31 patients with AA included from the E1201 and E1202 studies were analyzed for cytomorphologic changes in bone marrow. Twenty-one patients with AA who were refractory or intolerant to IST (15 with NSAA and 6 with SAA) were treated with eltrombopag in the E1201 study, and 10 patients with AA who were IST naive were treated with eltrombopag in combination with rabbit ATG/CsA in the E1202 study Table 2.

**Table 2:** Patient Characteristics and Morphologic Evaluation at Screening

	E1201 Study (n = 21)^a^	E1202 Study (n = 10)^b^
**Patient characteristics**		
Median age (range), y	53 (19-79)	55.5 (39-67)
Median time since diagnosis (range), mo	75 (1-297)	17.7 (3-102)
Severity, No. (%)	Nonsevere, 6 (29)	Stage II (moderate), 2 (20)
	Severe, 15 (71)	Stage III (moderately severe), 1 (10)
		Stage IV (severe), 3 (30)
		Stage V (very severe), 4 (40)
**Morphologic evaluation at screening, No.**		
Dysgranulopoiesis		
Evaluated	19	7
Dysgranulopoiesis ≥10%	0	0
Dysgranulopoiesis <10%	19	7
Could not be evaluated	2	3
Dyserythropoiesis		
Evaluated	14	6
≥10% at screening	6 (1 ring sideroblasts + <5% dyserythropoiesis at screening)	1
<10% at screening	8	5
Could not be evaluated	7	4
Dysmegakaryopoiesis		
Evaluated	1	0
Presence of dysplastic form	0	0
Absence of dysplastic form	1	0
Could not be evaluated	20	10

^a^E1201 patients 002, 003, 004, 006, 007, 011, 012, 017, 019, 022, 023, 028, 031, 032, 036, 037, 042, 051, 052, 053, and 057.

^b^E1202 patients 106, 111, 116, 117, 118, 121, 131, 146, 147, and 149.

### Hematologic Improvement

Of the 21 patients included from the E1201 study, 10 (48%) achieved hematologic responses in at least 1 lineage at 6 months after the start of treatment. Five patients achieved bilineage responses, and 1 patient achieved a trilineage response. In the 10 patients included from the E1202 study, the ORR with eltrombopag in combination with rabbit ATG/CsA at week 26 was 70.0%.

### Morphologic Findings

#### Blasts in Peripheral Blood and Bone Marrow After the Start of Eltrombopag Treatment

**Figure 2:**
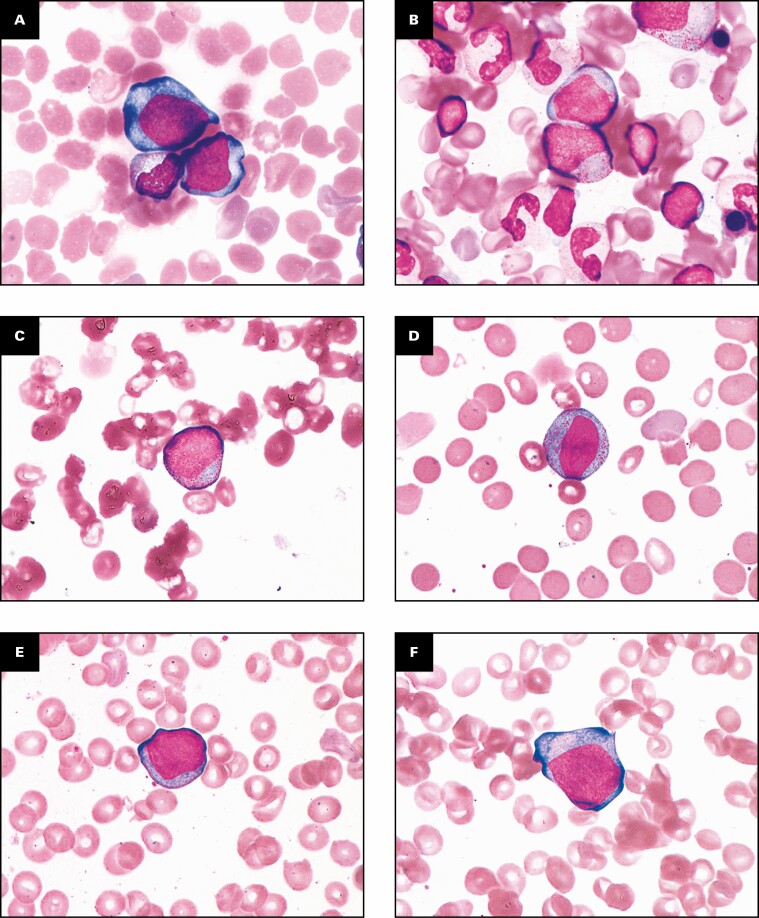

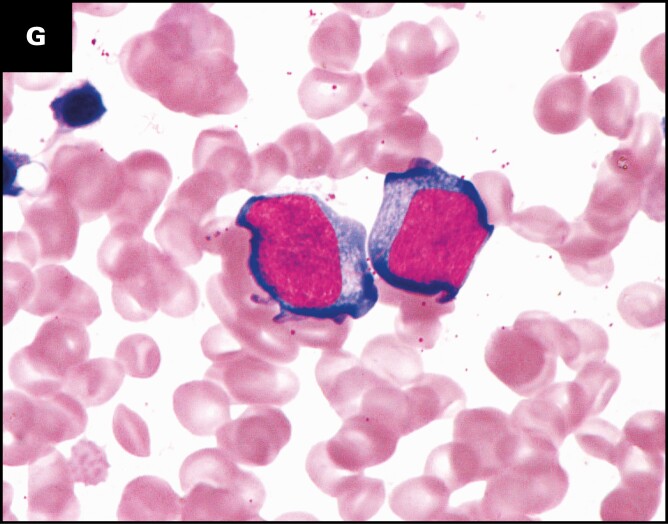
Increased blast count in bone marrow after the start of eltrombopag treatment. **A**, Study E1201 patient 003 at week 130 (May-Grünwald-Giemsa, ×1,000). **B**, Study E1201 patient 019 at week 26 (May-Grünwald-Giemsa, ×1,000). **C**, **D**, Study E1201 patient 028 at week 52 (May-Grünwald-Giemsa, ×1,000). **E**, **F**, Study E1202 patient 146 at week 14 (May-Grünwald-Giemsa, ×1,000). **G**, Study E1202 patient 147 at week 26 (May-Grünwald-Giemsa, ×1,000).

**Figure 3:**
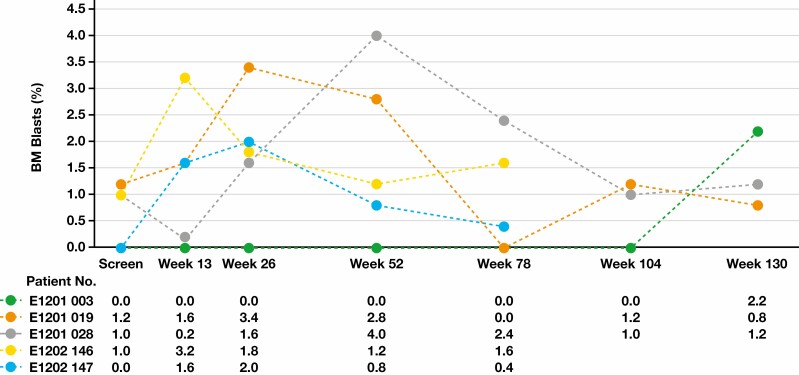
Change in bone marrow (BM) blast count (patient-level data). An increase in BM blasts was defined as a BM blast count more than double that at the time of screening. If the blasts were <1.0% at the time of screening, the increase in BM blasts was defined as >2%.

None of the patients showed blasts in peripheral blood at screening and after the start of eltrombopag treatment. In contrast, in 5 patients (E1201 [patients 003, 019, and 028]; E1202 [patients 146 and 147]), the bone marrow blast count increased after the start of treatment as compared with screening values Figures 2 and 3, Table 3. All patients with increased bone marrow blast count compared with the time of screening had higher marrow cellularity than at screening. According to the WHO classification,^16^ the cutoff value of the bone marrow blast count of MDS with excess blast is 5%. In this study, however, the increase in bone marrow blasts was transient in all 4 patients (019, 028, 146, and 147) who underwent bone marrow examinations at follow-up, while the bone marrow blast count was reportedly less than 5%.

**Table 3:** Relationships Between Morphologic Findings and Hematologic Improvement

E1201 Study^a^								
Patient No.			003	007	011	019	028	057
		Observed Timing of Increase in Bone Marrow Blasts	Week 130	Not Observed	Not Observed	Week 26, Week 52	Week 52, Week 78	Not Observed
Cytomorphologic Findings After Eltrombopag Treatment		Observed Timing of Dysmegakaryopoiesis	Week 130	Week 130	Week 13	Week 26 (≥10%), Week 52	Not Observed	Week 130
**Hematologic response** ^b^	Week 26	Er-R	+	+	nd	+	+	+
		Ne-R	−	−	nd	−	+	−
		Pl-R	−	+	nd	−	+	+
	Week 52	Er-R	+	+	nd	+	+	+
		Ne-R	−	+	nd	+	−	−
		Pl-R	−	+	nd	−	+	+
	Week 78	Er-R	+	+	nd	+	+	+
		Ne-R	−	−	nd	−	+	+
		Pl-R	−	+	nd	+	+	+
	Week 104	Er-R	+	+	nd	+	+	+
		Ne-R	−	−	nd	+	+	−
		Pl-R	−	+	nd	+	+	+
	Week 130	Er-R	+	−	nd	+	+	+
		Ne-R	+	−	nd	−	+	+
		Pl-R	−	+	nd	+	+	+
	F/U week 26	Er-R	nd	nd	−	nd	nd	nd
		Ne-R	nd	nd	na	nd	nd	nd
		Pl-R	nd	nd	−	nd	nd	nd
**E1202 Study** ^c^								
**Patient No.**			**106**		**117**		**146**	**147**
**Cytomorphologic Findings After Eltrombopag Treatment**		Observed Timing of Increase in Bone Marrow Blasts	Not Observed		Not Observed		Week 14	Week 26
		Observed Timing of Dysmegakaryopoiesis	Week 26 (≥10%), Week 52, Week 78		Week 26 (≥10%)		Week 14, Week 78	Not Observed
**Hematologic response** ^b^	Week 14	Er-R	−		−		−	−
		Ne-R	+		+		−	−
		Pl-R	−		−		+	−
	Week 26	Er-R	+		+		−	+
		Ne-R	+		+		+	+
		Pl-R	+		+		+	+
	Week 52	Er-R	+		−		−	+
		Ne-R	+		+		+	+
		Pl-R	+		+		+	+
	Week 78	Er-R	+		−		−	+
		Ne-R	+		+		+	+
		Pl-R	+		−		+	+
	F/U week 26	Er-R	nd		nd		nd	nd
		Ne-R	nd		nd		nd	nd
		Pl-R	nd		nd		nd	nd

F/U, follow-up; Er-R, erythroid response; na, not applicable; nd, test not performed; Ne-R, neutrophil response; Pl-R, platelet response.

^a^E1201 study: The last bone marrow examination during eltrombopag administration for patients 003, 007, 019, 028, and 057 was week 130. The last bone marrow examination during eltrombopag administration for patient 011 was week 13. Bone marrow examination after discontinuing eltrombopag administration was done at week 26 in patient 011.

^b^Based on the response criteria in Table 1, + indicates a positive hematologic response and − indicates a negative hematologic response.

^c^E1202 study: The last bone marrow examination during eltrombopag administration for patients 106, 117, 146, and 147 was week 78. In patient 011, eltrombopag was discontinued because of insufficient hematologic improvements at 3 months. Except for patient 011, some hematologic improvements were observed in patients in whom a transient increase in bone marrow blast count or dysplastic forms of megakaryocytes were observed.

#### Dysmegakaryopoiesis After the Start of Eltrombopag Treatment

**Figure 4:**
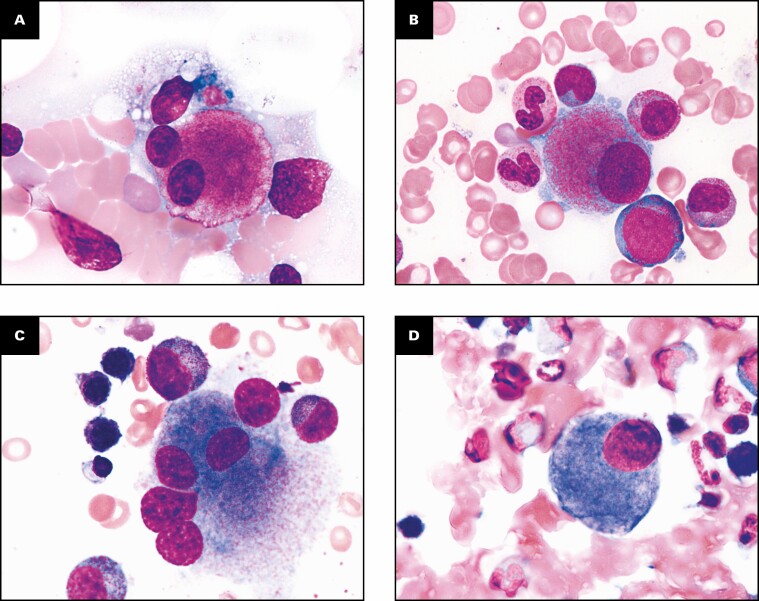

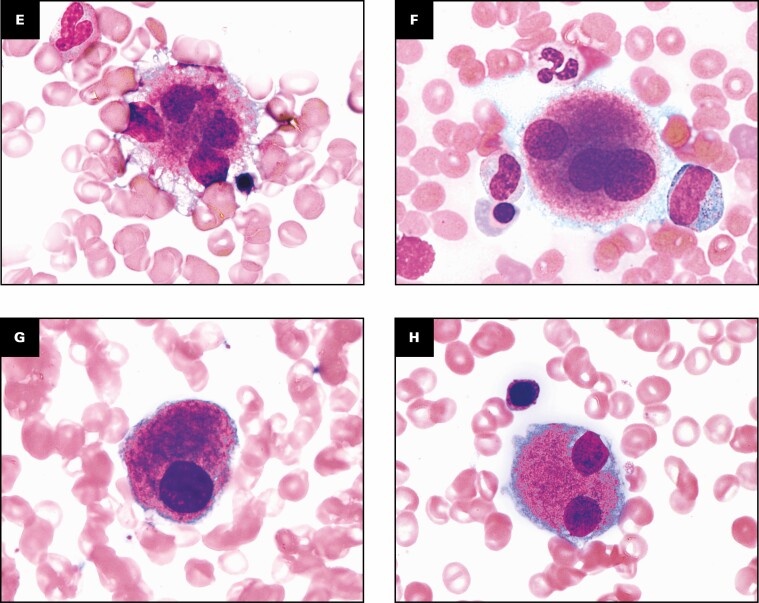
Dysmegakaryopoiesis after the start of eltrombopag treatment. **A**, Study E1201 patient 003 at week 130, multinucleated megakaryocyte (May-Grünwald-Giemsa, ×1,000). **B**, Study E1201 patient 007 at week 130, megakaryocyte with a nonlobulated nucleus (May-Grünwald-Giemsa, ×1,000). **C**, Study E1201 patient 011 at week 13, multinucleated megakaryocyte (May-Grünwald-Giemsa, ×1,000). **D**, Study E1202 patient 019 at week 26, megakaryocyte with a nonlobulated nucleus; dysmegakaryopoiesis ≥10% was found at week 26. Dysmegakaryopoiesis ≥10% was not found from week 52 (May-Grünwald-Giemsa, ×1,000). **E**, Study E1201 patient 057 at week 130, multinucleated megakaryocyte (May-Grünwald-Giemsa, ×1,000). **F**, Study E1202 patient 106 at week 26, multinucleated megakaryocyte; dysmegakaryopoiesis ≥10% was found at week 26; dysmegakaryopoiesis ≥10% was not found from week 52 (May-Grünwald-Giemsa, ×1,000). **G**, Study E1202 patient 117 at week 26, megakaryocyte with a nonlobulated nucleus; dysmegakaryopoiesis ≥10% was found at week 26; dysmegakaryopoiesis ≥10% was not found from week 52 (May-Grünwald-Giemsa, ×1,000). **H**, Study E1202 patient 146 at week 78, multinucleated megakaryocyte (May-Grünwald-Giemsa, ×1,000).

In 30 of 31 patients, the morphology of megakaryocytes could not be evaluated based on the small number of megakaryocytes in the bone marrow at screening. Only 1 patient (patient 012 from the E1201 study) was eligible for evaluation of the morphologic findings in the megakaryocytic lineage and did not have dysplastic forms at screening. In 8 patients (E1201 [patients 003, 007, 011, 019, and 057]; E1202 [patients 106, 117, and 146]), dysplastic forms of megakaryocytes were found in the bone marrow after the start of eltrombopag treatment Figure 4, Table 3. These patients showed increased numbers of megakaryocytes compared with the time of screening. The detected dysplastic forms of megakaryocytes were mostly megakaryocytes with a hypolobulated or nonlobulated nucleus. Multinucleated megakaryocytes were found, but the ratio was low. In addition, no micromegakaryocytes were found. Dysmegakaryopoiesis of 10% or more was found in 3 patients (patients 019, 106, and 117). The detected dysplastic forms were transient in 2 patients (patients 019 and 117).

#### Dyserythropoiesis ≥10% After the Start of Eltrombopag Treatment

Eleven patients (E1201 [patients 004, 006, 023, 031, 032, 051, and 053]; E1202 [patients 111, 121, 131, and 147]) could not be evaluated for morphologic findings in erythroid lineage in the bone marrow at screening. AA with dyserythropoiesis of 10% or more in the bone marrow at screening was found in 7 patients (E1201 [patients 003, 007, 011, 012, 019, and 037]; E1202 [patient 149]). Most of the detected dysplastic forms of erythroblasts were megaloblastic changes. Only 1 patient (patient 012 from the E1201 study) had ring sideroblasts in the bone marrow at screening, but the ratio was less than 5%. Among 7 patients with dyserythropoiesis of 10% or more in the bone marrow at screening, dyserythropoiesis of 10% or more was persistently observed in the bone marrow after the start of treatment in 4 patients (patients 007, 011, 012, and 037 from the E1201 study). In 13 patients with dyserythropoiesis of less than 10% in the bone marrow at screening, dyserythropoiesis of 10% or more in the bone marrow was found after the start of treatment in 5 patients (E1201 [patients 002, 022, 028, and 042]; E1202 [patient 106]). The detected dysplastic forms of erythroblasts were mostly megaloblastic changes, and ring sideroblasts were not observed in these 5 patients.

#### Dysgranulopoiesis ≥10% After the Start of Eltrombopag Treatment

In 5 patients (E1201 [patients 006 and 023]; E1202 [patients 111, 121, and 131]) at screening, dysgranulopoiesis could not be evaluated because of the small number of granulocytes. In the remaining 26 patients with AA at screening, dysgranulopoiesis of 10% or more was not found in the bone marrow. No patient showed dysgranulopoiesis of  10% or more after the start of treatment.

### Relationships Between Morphologic Findings and Hematologic Improvement

The bone marrow cellularity increased compared with the time of screening in 11 patients in the E1201 study and 9 patients in the E1202 study. In most of the 20 patients, the bone marrow cellularity increased from hypoplasia (5%-19%) to slight hypoplasia (20%-29%) or normoplasia (30%-59%). In the patients with increased bone marrow cellularity, hematologic response was observed in at least 1 lineage in 9 patients in the E1201 study and 7 patients in the E1202 study. Increased numbers of megakaryocytes compared with the time of screening were observed in 6 patients in the E1201 study and 7 patients in the E1202 study. In most of these 13 patients, the numbers of megakaryocytes increased from marked decrease (no megakaryocytes) to decrease (<0.5/HPF) or slight decrease (0.5-0.9/HPF). Trilineage hematologic response was observed in all patients with increased numbers of megakaryocytes.

Increased blasts in bone marrow or dysmegakaryopoiesis after the start of treatment were observed in 10 patients (increased blasts in bone marrow and dysmegakaryopoiesis [patients 003, 019, and 146]; isolated increased blasts in bone marrow [patients 028 and 147]; isolated dysmegakaryopoiesis [patients 007, 011, 057, 106, and 117]). Nine of these 10 patients (except for patient 011 from the E1201 study) showed hematologic improvement in 1 or more lineages Table 3.

In patient 011, dysplastic forms of megakaryocytes were found in the bone marrow at week 13 after eltrombopag treatment Table 3. In this patient, the response at 3 months was insufficient; therefore, eltrombopag was discontinued. Cytogenetic abnormality (trisomy 8) developed at 6 months after the cessation of eltrombopag. This patient had dyserythropoiesis of 10% or more in the bone marrow at screening. The patient also had dyserythropoiesis of 10% or more in the bone marrow when the cytogenetic abnormality was detected. Because of the small number of megakaryocytes, however, it was not possible to assess the presence of megakaryocyte dysplasia in bone marrow either at screening (1 megakaryocyte/2 smears) or when chromosomal abnormalities were observed (2 megakaryocytes/2 smears). Other patients with increased bone marrow blasts and/or dysmegakaryopoiesis after treatment initiation showed no chromosomal abnormalities. In addition, in all patients of the E1201 and E1202 studies, FISH analysis did not detect the loss of chromosome 7 at the end of the observation period.

Of the 7 patients with dyserythropoiesis of 10% or more in the bone marrow at screening, 4 patients reported persistent dyserythropoiesis of 10% or more from screening to after treatment. Other than patient 011 in the E1201 study, 6 patients with dyserythropoiesis of 10% or more in the bone marrow at screening showed hematologic improvement in 1 or more lineages until at least 26 weeks after the administration of eltrombopag (best response: 4 trilineages [patients 007, 019, 037, and 149]; 1 bilineage [patient 003]; 1 unilineage [patient 012]). Patient 011 showed no response.

Among 13 patients with dyserythropoiesis of less than 10% in the bone marrow at screening, 5 had dyserythropoiesis of 10% or more in the bone marrow after treatment initiation. Of these 5 patients, 1 (patient 002) showed a bilineage response and 2 (patients 028 and 106) showed a trilineage hematologic response, with erythroid hyperplasia in 1 (patient 106).

In the E1201 study, marrow fibrosis (grade 1 or 2 according to the European Consensus Scale [ECS]) was observed in 2 patients (patients 032 and 036) after administration. No patients showed increased bone marrow blast count and/or dysmegakaryopoiesis, and there was no hematologic improvement in these 2 patients. In the E1202 study, marrow fibrosis (grade 1 according to the ECS) was observed in 2 patients (patients 111 and 147) after administration. Increased bone marrow blast count was observed in 1 (patient 147) of 2 patients. Neither of these 2 patients showed dysmegakaryopoiesis, although both showed trilineage hematologic improvement.

## DISCUSSION

AA is a hypocellular bone marrow disorder often characterized by reduced or no erythropoiesis. Although common, dyserythropoiesis alone cannot distinguish AA from MDS. Dysplasia of megakaryocytes, granulocytic cell lineages, and blasts in the peripheral blood, which are the characteristic bone marrow features in MDS, are not seen in AA.^22^ Dyserythropoiesis is found in MDS but is unsuitable as an indicator of clonal evolution.^23^

In the present study involving 31 patients with AA, 20 could be evaluated for dyserythropoiesis, of whom 7 showed dyserythropoiesis of 10% or more; only 1 could be evaluated for megakaryopoiesis but had no dysplastic megakaryocyte form; and 26 could be evaluated for dysgranulopoiesis, of whom none had dysgranulopoiesis of 10% or more in the bone marrow at screening. After eltrombopag administration, an increase in bone marrow blast count or dysplastic forms of megakaryocytes were observed in patients with AA from both studies (E1201, 6 patients; E1202, 4 patients), although the bone marrow blast count was less than 5%. In all 4 patients who could be followed, the increased bone marrow blast count was transient. Evaluation of dysplastic forms in a megakaryocytic lineage is important in the diagnosis of MDS and AML; micromegakaryocytes have a high specificity in myeloid neoplasms, and the presence of micromegakaryocytes is a specific characteristic of MDS and AML. The diagnostic basis of MDS for dysplastic forms of megakaryocytes other than micromegakaryocytes is not so specific.^24^ In the present analysis, micromegakaryocytes were not found in any patient with AA.

In a phase 2 study of 25 patients with AA refractory to immunosuppression, clonal evolution or cytogenetic abnormality associated with dysplasia, including the loss of chromosome 7, was reported in 2 patients who did not respond to treatment with eltrombopag.^9^ In the present analysis of patients with AA from the E1201 and E1202 studies, no abnormality of chromosome 7 and no evolution to MDS and AML were observed. The EQoL-MDS phase 2 superiority study (ClinicalTrials.gov identifier NCT02912208) in adult patients with lower-risk MDS and severe thrombocytopenia reported that AML evolution or disease progression occurred in 7 of 59 (12%) patients in the eltrombopag group vs 5 of 31 (16%) patients in the placebo group (χ ^2^ = 0.06, *P* = .81).^25^ In a phase 2 dose-modification study to investigate the safety and efficacy of eltrombopag in 25 patients with lower-risk MDS, 6 patients had disease progression without any association with the expansion of mutated clones, and none progressed to develop AML during the study.^17^ In an open-label, phase 3, randomized trial of horse ATG and CsA with or without eltrombopag in naive patients with severe or very severe AA (ClinicalTrials.gov identifier NCT02009747), high-sensitivity next-generation sequencing analysis showed no difference in terms of somatic myeloid mutations between the 2 groups at 6-month follow-up.^18^ Thus, the role of eltrombopag in clonal evolution is still unclear. In the E1201 and E1202 studies demonstrated hematologic improvement in at least 1 lineage without cytogenetic abnormalities was reported in all patients in whom an increase in bone marrow blast count or dysplastic forms of megakaryocytes was observed, except in 1 patient (patient 011) who discontinued eltrombopag treatment because of insufficient response at 3 months. Thus, these morphologic findings during eltrombopag administration may be signs of recovery of hematopoiesis rather than progression to clonal disease. In general, dysmegakaryopoiesis and an increase in bone marrow blast count are morphologic findings indicative of clonal evolution. To rule out that these morphologic changes may be associated with clonal or neoplastic abnormalities, bone marrow aspiration available from the study patients after discontinuation of eltrombopag treatment is necessary. Except for patient 001, however, these aspirations were not done. Six patients had hematologic improvements before these morphologic findings had been identified. Therefore, lack of increased blasts or dysmegakaryopoiesis does not necessarily indicate the absence of hematologic response.

In a phase 1/2 dose-escalation clinical study in 44 patients with lower-risk MDS and thrombocytopenia, monotherapy with romiplostim (a TPO receptor agonist) achieved a durable platelet response in 19 (46%) patients, although 2 patients had an increase in bone marrow blast count and progressed to AML and another 4 patients experienced a transient increase in bone marrow blast count. These results raised concerns about a possible association of romiplostim treatment with leukemic progression. Therefore, the study was prematurely discontinued because of a suspected safety concern for progression to AML or an incorrect diagnosis or treatment for AML.^26^ The 5-year follow-up data, however, showed that the risk of leukemic progression was not significantly different between romiplostim and placebo.^27^ These findings suggested that the increase in bone marrow blast count during romiplostim treatment may not be related to clonal evolution. Similarly, in the present analysis, a transient increase in bone marrow blast count and dysmegakaryopoiesis during eltrombopag treatment may not be signs of transformation to AML or MDS. These findings may lead to incorrect diagnoses of myeloid malignancies.

Dysmegakaryopoiesis without micromegakaryocytes and a transient increase of less than 5% in bone marrow blast count are a laboratory pattern that may be a clinical marker of hematologic improvement with eltrombopag for patients with AA. Therefore, even if these findings were confirmed in patients with AA receiving eltrombopag therapy, physicians do not have to be concerned about the evolution to AML or MDS. Careful clinical follow-up and tests for detection of driver mutations for AML or MDS are required.

## CONCLUSIONS

In general, dysmegakaryopoiesis and an increase in bone marrow blast count are morphologic findings indicative of clonal evolution; however, the cytomorphologic changes in bone marrow during eltrombopag administration are unknown. The results of the present study suggest that dysmegakaryopoiesis and transient mild increases in bone marrow blasts in patients with AA treated with eltrombopag may be signs of hematologic improvement and not portend clonal evolution. Cytogenetic and molecular data are necessary, however, in AA following eltrombopag.

## Notes

Funding: This work was supported by GlaxoSmithKline; however, as of March 2, 2015, eltrombopag is an asset of Novartis Pharma.
